# Willingness to pay for chronic disease management services provided by primary care nurses

**DOI:** 10.1186/s12960-024-00935-8

**Published:** 2024-07-08

**Authors:** HanNah Park, JuYoung Yoon

**Affiliations:** 1 College of Nursing, Seoul National University, 103 Daehak-Ro, Jongno-Gu, Seoul, 03080 Republic of Korea; 2 Research Institute of Nursing Science, Seoul National University, Seoul, 03080 Republic of Korea

**Keywords:** Contingent valuation method, Primary care, Primary care nurses, Willingness to pay

## Abstract

**Background:**

Due to the rapid increase in chronic diseases in South Korea, the Korean government has expanded chronic disease management to primary care. Thus, the role of primary care nurses is critical. However, the fee for chronic disease management services provided by primary care nurses has not been set, and few studies have evaluated the value of nursing services. This study aimed to estimate the willingness to pay (WTP) for chronic disease management services provided by primary care nurses and to identify the factors that affect WTP.

**Methods:**

This study adopted a descriptive research design and conducted a cross-sectional online survey from January 16 to 18, 2023. The inclusion criteria were community residents aged ≥ 20 years living in South Korea and capable of participating in online surveys. A total of 520 people participated in this study. A contingent valuation method (CVM) was used with double-bound dichotomous choice questions along with open-ended questions. The mean WTP was calculated using a Tobit model.

**Results:**

The mean WTP of the 520 study participants for one chronic disease management service provided by primary care nurses was 15,390.71 Korean won ($11.90). Factors affecting WTP were having a chronic disease, recognition of primary care nurses, and the first-bid price. Community residents with fewer chronic diseases, high awareness of primary care nurses, and a higher first-bid price showed higher WTP for chronic disease management services provided by primary care nurses.

**Conclusions:**

Primary care is important worldwide due to the increasing number of chronic diseases, and Korea is no exception. However, payment for services by primary care nurses is undervalued compared to their critical role and skills. This has led to problems such as a primary care nurse shortage and burnout. This study estimated individuals’ WTP for chronic disease management services provided by primary care nurses. The results can be used as a basic resource for setting the fee for services provided by primary care nurses. It is also a good starting point to understand the benefits of primary care nurse services.

**Supplementary Information:**

The online version contains supplementary material available at 10.1186/s12960-024-00935-8.

## Background

Chronic diseases are a leading cause of death and disability worldwide [1]. As a result, the importance and need for primary care to effectively manage chronic diseases has greatly increased [1, 2]. Barbara Starfield defined primary care as “the first entry into the healthcare system that focuses on people, not diseases over a long period of time, provides services for all diseases except very rare ones, and coordinates and integrates services provided elsewhere by other providers” [3]. Globally, investments in primary care have improved access to healthcare, equity, quality of care, and health outcomes [2].

In South Korea (Korea hereafter), although the importance of primary care has been emphasized due to the increasing number of people with chronic diseases [4], Korea’s healthcare system is fragmented and lacks continuity. Moreover, the roles of primary care clinics and hospitals are mixed and the role of primary care providers in chronic disease management is unknown. This lack of clear boundaries has led to a hospital-centered healthcare system in Korea. For example, many patients with mild symptoms use hospitals rather than local clinics, and the average length of a hospital stay is twice the average of Organization for Economic Co-operation and Development (OECD) countries. As a countermeasure, OECD recommended that Korea establish a strong community-based primary care system [5].

Many countries have recognized the importance of a team-based approach to effectively manage chronic diseases in primary care [2]. In particular, nurses play a key role in multidisciplinary teams that provide primary care, and it would be difficult to provide adequate care for multiple patients without their care. Introducing primary care nurses in local clinics has improved patients’ physical and mental health status, quality of life, and satisfaction and has enhanced the quality of the services [6].

In Korea, local clinics are primarily staffed by certified nursing assistant. As of 2020, only 6.5% of nurses worked in local clinics [7]. The Korean government has recently introduced nurses into primary care to improve care and particularly to manage chronic diseases. However, the payment for the services provided by primary nurses is undervalued compared to the importance of their role and the skills in primary care. This unrecognized value has led to a shortage of primary care nurses and burnout [8]. Establishing a fee for primary care nurse services would promote employment for primary care nurses at local clinics and reflect the value they provide in primary care in clinics [8, 9].

This study used the contingent valuation method (CVM) to estimate community members’ willingness to pay (WTP) for primary care nurse services. The CVM approach involves presenting a hypothetical scenario to respondents and measuring their WTP, including the value of the goods. Specifically, they respond to a survey after reading the hypothetical scenario and assuming that the condition exists in the market. This approach can confirm the preference and value individual consumers attach to the services [10]. It also allows for direct comparison between services since the benefits obtained through the services are measured in the same monetary unit. The results are useful for evaluating the effectiveness of a business [11]. The value consumers assign to services plays an essential role in determining the price and consumption of services [12]. In this respect, researchers have argued that determining the cost for nursing services based on consumers’ reported value for the services is more convincing as a policy [10].

Despite the advantages of CVM, it can cause bias because the WTP measurement uses only hypothetical scenarios and surveys [13]. Furthermore, there are significant differences in recognizing benefits among respondents, and WTP is affected by respondents’ general and socioeconomic characteristics [10]. To derive WTP more accurately, it is necessary to also examine factors that affect WTP [14].

Thus, this study aimed to estimate WTP for chronic disease management services provided by primary care nurses in South Korea using CVM and to identify factors affecting WTP.

## Methods

### Study design

**Fig. 1:**
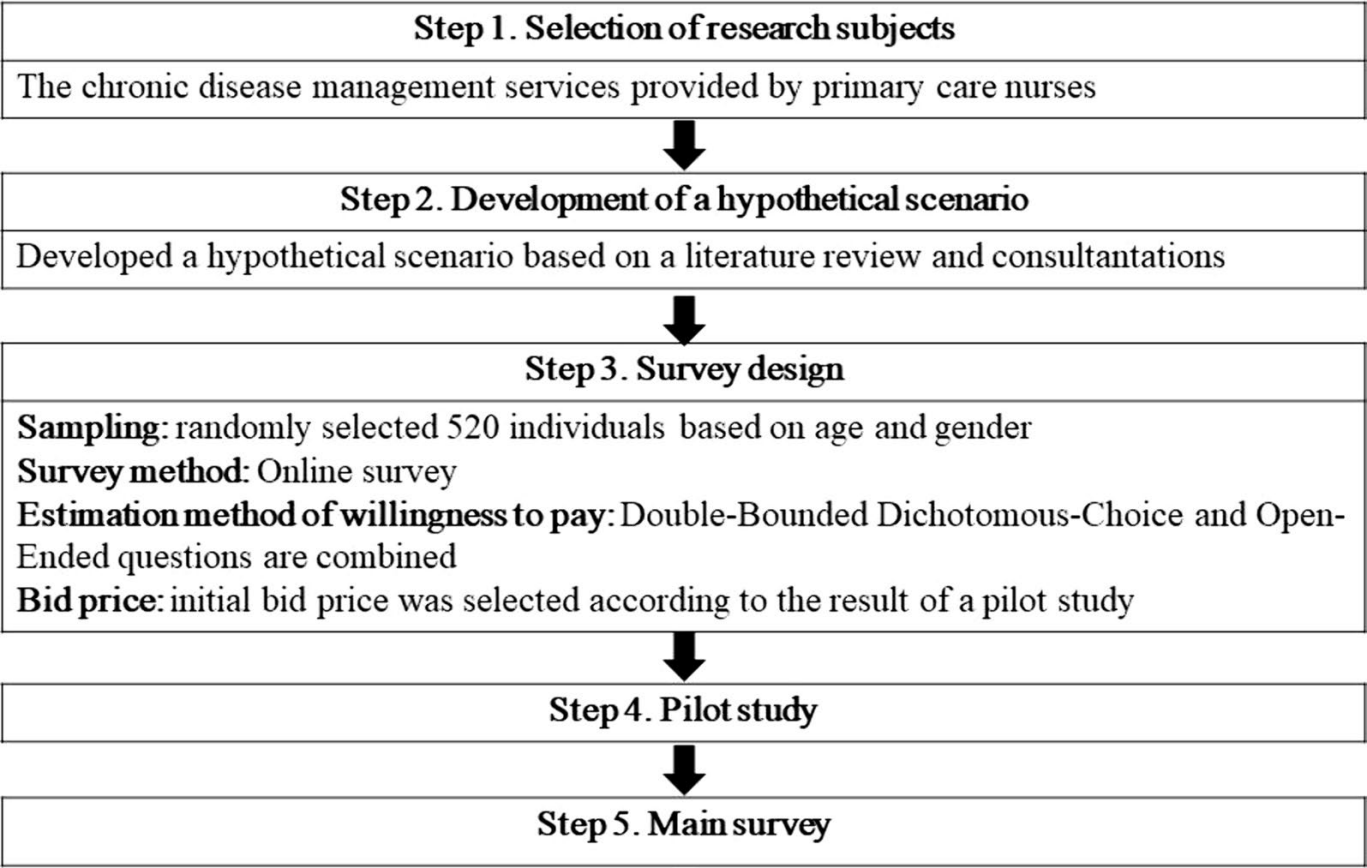
Study process diagram

This study adopted a descriptive cross-sectional research design. CVM with double-bounded dichotomous choice (DBDC) questions along with open-ended questions was used to estimate participants’ WTP for chronic disease management services provided by primary care nurses. Figure 1 shows the procedure of this study.

### Participants

Prior studies have recommended recruiting > 500 individuals to estimate WTP [15]. Thus, this study selected 520 individuals from all regions throughout Korea using the quota sampling method with convenience sampling. The study applied the quota sampling method by gender (males and females) and age group (20–29, 30–39, 40–49, 50–59, and ≥ 60 years).

The inclusion criteria for this study were as follows: Korean community residents (1) aged ≥ 20 years living in Korea; (2) those who could understand and respond to the questionnaires, and (3) those who agreed to participate in this study. The exclusion criterion for the main study was individuals who could not participate in online surveys (e.g., had no access to the Internet).

### Bid price

The initial bid price presented to participants can affect WTP [16]. In this study, to reduce bias, the initial bid price was selected based on the results of a pilot study. The initial bid price was set within the range of 15–85% of the WTP results from the pilot study [17]. Presenting four to six bid prices is recommended since presenting too many or too few bid prices can decrease the model fitness [18]. Therefore, in this study, four initial bid prices were selected based on the cumulative percentages of 15%, 50%, 75%, and 85% (1500 Korean won (KRW), 5000 KRW, 10,000 KRW, and 25,000 KRW, respectively).

### Data collection

The pilot study was conducted with 25 individuals using CVM with open-ended questions via telephone surveys from January 2 to 15, 2023. Based on the results of the pilot study, a reasonable range for bid prices was determined and four bid prices were set. The main online survey was conducted from January 16 to 18, 2023 by a major online survey company (EMBRAIN). This online survey company has a large number of panels in Korea (> 1.7 million) and has conducted surveys for many small- and large-scale research projects. ISAS software, developed by EMBRAIN, was utilized for the survey. To confirm that the online survey was conducted as intended based on the study design, the researcher verified the accuracy of the survey form and content through a test link before launching the actual online survey. To prevent duplicate survey responses, the parameters were set so each participant could only respond to the survey once per IP.

### Measures

The questionnaire included 24 questions, which took approximately 15–20 min to complete. Before completing the survey, participants were presented with a hypothetical scenario and were asked to imagine themselves in this scenario. Then, participants answered 4 items to estimate their WTP and 19 items about factors that affect WTP. The questionnaire was divided into four versions, each corresponding to one of the four bid prices (1500 KRW, 5000 KRW, 10,000 KRW, and 25,000 KRW) determined in the pilot study. These versions were randomly assigned to the study participants, with 130 participants assigned to each version, totaling 520 participants.

### Hypothetical scenario

The researchers developed a hypothetical scenario based on previous studies (see Additional file 1 for the scenario) [10, 13, 19:25]. The scenario was then reviewed by three nurse care coordinators who worked at a local clinic. Their feedback on patient characteristics and home environments made the hypothetical scenario more realistic.

### WTP

We used two types of questions to measure participants’ WTP. We first asked a double-bounded dichotomous choice (DBDC) questions and a follow-up question depending on the respondent’s answer. They were then asked an open-ended question and a follow-up question depending on the respondent’s answer (see Additional file 2 for a complete description).

*Initial DBDC questions.* Participants’ WTP for chronic disease management services provided by primary care nurses was measured using a series of questions. First, we asked, “Would you be willing to pay (first-bid price) for chronic disease management services provided by primary care nurses?” There were two responses: “yes” and “no.” If the response was “yes” for the first-bid price, the second-bid price was double the first-bid price. If the response was “no” for the first-bid price, the second-bid price was half of the first-bid price. Next, we asked participants, “Would you be willing to pay (second-bid price) for chronic disease management services provided by primary care nurses?” There were two responses: “yes” and “no.” We asked the same follow-up question as stated above based on their answer. For these DBDC questions, four responses were possible: “yes–yes,” “yes–no,” “no–yes,” or “no–no.”

*Open-ended questions.* We then asked an open-ended question if the response was “yes–yes,” “yes–no,” or “no–yes,” “How much are you willing to pay for chronic disease management services provided by primary care nurses?” The answer to this open-ended question was their WTP for chronic disease management services provided by primary care nurses. If the response was “no–no,” we asked the participant a follow-up question, “Are you unwilling to pay any amount for this service?” If the response was “no,” (indicating that they would be willing to pay something) we asked one more open-ended question, “How much are you willing to pay for chronic disease management services provided by primary care nurses?” If the response was “yes,” (indicating that they were unwilling to pay any amount) their WTP was recorded as zero, suggesting that the participant was not willing to pay any amount for such services.

### Factors that affect WTP

Based on a literature review and consultation with three nurse care coordinators working in Korea, we determined factors that could affect WTP for chronic disease management services provided by primary care nurses. The following factors were selected: gender [19, 20], age [20, 21], residence [22], education level [19, 23, 24], monthly household income [10, 19, 21, 23, 25], marital status [25], whether the respondent is currently working [10, 22], subjective health status [22], chronic disease [25], social activity [22], recognition of primary care nurses [23], recognition of primary care pilot program for chronic disease management [23], and first bid [13].

### Statistical analysis

The collected data were analyzed using Statistical Package for the Social Sciences, version 26.0, and STATA, version 17. General characteristics were presented as frequencies, percentages, means, and standard deviations. The Tobit model, which is appropriate to estimate censored data, was applied because the collected data in this study were left-censored with the lower limit of WTP fixed to 0. Thus, the Tobit model was used to estimate WTP and determine the factors affecting WTP for chronic disease management services provided by primary care nurses [26].

## Results

### General characteristics

The general characteristics of the study participants are described in Table 1. A total of 520 people participated in this study, with a mean age of 44.13 ± 13.84 years. The majority resided in a metropolitan city (n = 279, 53.7%), held a 4-year college degree or higher (n = 387, 74.4%), and reported an average monthly household income of 4,000,000 Korean won or more (n = 259, 49.8%). While the majority of the participants were aware of the primary care pilot program for chronic disease management (n = 437, 84.0%), only a quarter of participants were aware that the program used primary care nurses to deliver the services (n = 130, 25%).

**Table 1:** General characteristics of the study participants

(N = 520)				
Variables	Categories	Total, n (%)	M ± SD	Range
Gender	Male	247(47.5)		
	Female	273(52.5)		
Age (year)	20–29	107 (20.6)		
	30–39	104 (20.0)		
	40–49	105 (20.2)		
	50–59	105 (20.2)		
	over 60	99 (19.0)	44.13 ± 13.84	20–72
Residence	Metropolitan city	279 (53.7)		
	City and Town	213 (41.0)		
	County	28 (5.4)		
Education level	Elementary school or under	1 (0.2)		
	Middle school	4 (0.8)		
	High school	128 (24.6)		
	College or higher	387 (74.4)		
Monthly household income (10,000 KRW)	< 100	35 (6.7)		
	100 to < 200	32 (6.2)		
	200 to < 300	90 (17.3)		
	300 to < 400	104 (20.0)		
	≥ 400	259 (49.8)		
Marital status	Married	291 (56.0)		
	Others*	229 (44.0)		
Type of housing	Homeowner	341 (65.6)		
	security deposit, monthly rent, others	179 (34.4)		
Living with family	Yes	431 (82.9)		
	No	89 (17.1)		
Currently working	Yes	371 (71.3)		
	No	149 (28.7)		
Type of health insurance	National health insurance	508 (97.7)		
	Medical benefit system	12 (2.3)		
Private health insurance	Yes	419 (80.6)		
	No	101 (19.4)		
Subjective health status	Bad	62 (11.9)		
	Average	221 (42.5)		
	Good	237 (45.6)		
EQ-5D	Good (> 0.8)	492 (94.6)		
	Bad (≤ 0.8)	28 (5.4)	0.88 ± 0.05	0.29–0.91
Chronic disease	Yes	230 (44.2)		
	No	290 (55.8)	0.61 ± 0.83	0–6
Current medication	Yes	174 (33.5)		
	No	346 (66.5)		
Social activity	Yes	247 (47.5)		
	No	273 (52.5)		
Health facility visited within a month	Yes	382 (73.5)		
	No	138 (26.5)		
Recognition of primary care nurses	Yes	130 (25.0)		
	No	390 (75.0)		
Recognition of primary care pilot program for chronic disease management	Yes	83 (16.0)		
	No	437 (84.0)		

M, mean; SD, standard deviation; KRW = Korean won; *Others = widowed, divorced, separated, never married

### Distribution of WTP responses

Table 2 shows the distribution of WTP responses for each bid price. As the bid price increased, participants tended to be less willing to pay for the services.

**Table 2:** Distribution of WTP responses

(N = 520)								
First-bid price	Second-bid price (higher)	Second bid price (lower)	Sample size, n	Acceptance (Yes) or Rejection (No) of the 1st and 2nd bids (1st–2nd), n (%)				
KRW (USD)*				Yes–Yes	Yes–No	No–Yes	No–No–No	No–No–Yes
1500 (1.16)	3000 (2.32)	750 (0.58)	130	83 (63.9)	20 (15.4)	10 (7.7)	3 (2.3)	14 (10.8)
5000 (3.86)	10,000 (7.73)	2500 (1.93)	130	55 (42.3)	47 (36.2)	13 (10.00)	5 (3.9)	10 (7.7)
10,000 (7.73)	20,000 (15.46)	5000 (3.86)	130	46 (35.4)	42 (32.3)	22 (16.9)	5 (3.9)	15 (11.5)
25,000 (19.32)	50,000 (38.65)	12,500 (9.66)	130	18 (13.9)	48 (36.9)	29 (22.3)	24(18.5)	11 (8.5)
Total			520	202 (38.8)	157 (30.2)	74 (14.2)	37 (7.1)	50 (9.6)

WTP, willingness to pay; KRW, Korean won; USD, United States dollar; *2022 (January 1, 2022 to December 31, 2022) yearly average exchange rate 1 USD = 1293.68 KRW. If the response was “yes” for the first bid, the second bid was double that of the first bid. If the response was “no” for the first bid, the second bid was half of the first bid

### Reasons for unwillingness to pay for chronic disease management services provided by primary care nurses

Table 3 shows the reasons for participants’ unwillingness to pay for chronic disease management services provided by primary care nurses. The main reason was “The country should provide this service for free” (n = 14, 28.0%), followed by “I am not interested in the chronic disease management services provided by nurses” (n = 10, 20.0%), and “I cannot afford to pay for this service” (n = 9, 18.0%).

**Table 3:** Reasons for unwillingness to pay for chronic disease management services provided by primary care nurses

(N = 50)	
Reasons	n (%)
I cannot afford to pay this service	9 (18.0)
I do not have time to get this service	3 (6.0)
I am not interested in the chronic disease management services provided by nurses	10 (20.0)
There are many alternative services around us that are similar to those services	7 (14.0)
The quality of the service is not reliable	3 (6.0)
Not enough information has been given about the service	2 (4.0)
The country should provide this service for free	14 (28.0)
It is not fair to ask me to pay for the service	2 (4.0)
I do not like this kind of question	0 (0.0)
Total	50 (100.0)

### Factors associated with WTP

Table 4 shows the results of the Tobit model with and without covariates. The model was statistically significant at the 1%significance level, and no multicollinearity was observed (max VIF = 1.67, mean VIF = 1.24). Three factors, chronic disease ($\beta$ < 0; *p* < 0.05), recognition of primary care nurses ($\beta$ > 0; *p* < 0.05), and the first-bid price ($\beta$ > 0; *p* < 0.001), were significantly associated with WTP. In other words, the fewer chronic diseases the participants reported, the greater the recognition of the need for disease management services provided by primary care nurses. In addition, the higher the first-bid price, the higher the WTP for chronic disease management services provided by primary care nurses.

**Table 4:** Tobit model with and without covariates for estimating WTP

Variables	Model without covariates		Model with covariates	
	Coefficient	t-value	Coefficient	t-value
Constant	13,137.04***	18.27	4242.12	0.64
Gender ref. female			139.58	0.10
Age (year)			− 425.28	− 0.69
Residence ref. metropolitan city			211.46	0.19
Education level ref. elementary school or lower			712.70	0.47
Monthly household income (10,000KRW)			1112.21	1.79
Marital status ref. not married			− 3888.83	− 1.90
Currently working			1498.48	1.04
Subjective health status			− 1857.06	− 1.36
Chronic disease			− 2882.51*	− 2.00
Social activity			1493.67	1.08
Recognition of primary care nurses			4731.96*	2.16
Recognition of primary care pilot program for chronic diseases management			1692.79	0.78
First bid ref. 1500KRW			0.70***	9.11
Number of observations	520		520	
Log likelihood	− 5304.70***		− 5254.05***	

WTP, willingness to pay; KRW, Korean Won; *p < 0.05, **p < 0.01, ***p < 0.001, ref., reference

### Estimated WTP

Table 5 shows the results for estimating the median WTP for chronic disease management services provided by primary care nurses using the Tobit model. The median WTP was 15,390.71 KRW ($11.90).

**Table 5:** Estimated WTP

Variables	Tobit model	
	Without covariate*	With covariates
Mean WTP	15,390.71 KRW $11.90	15,385.14 KRW $11.89
Number of observations	520	520
Log likelihood	− 5304.70	− 5254.05
*P*-value	< 0.001	< 0.001

WTP, willingness to pay; KRW, Korean Won; 2022 (January 1, 2022 to December 31, 2022) yearly average exchange rate 1 USD = 1293.68 KRW; *covariates = gender, age, residences, education level, monthly household income, marital status, currently working, subjective health status, chronic disease, social activity, recognition of primary care nurses, recognition of primary care pilot program for chronic disease management, first bid

## Discussion

The number of chronic disease patients has greatly increased globally, highlighting the importance of quality primary care [1, 2]. However, paying for such services is undervalued relative to the role and skills of primary care nurses. This lack of value has led to a nursing shortage and burnout among nurses [8, 9]. Thus, it is critical to estimate patients’ willingness to pay for services provided by primary care nurses. This study used CVM to estimate WTP for chronic disease management services provided by primary care nurses.

The estimated WTP for one chronic disease management service provided by primary care nurses in this study was 15,390.71 KRW ($11.90). One study reported that the mean consultation direct cost was $51.74 per service based on resource use, cost of follow-up consultations, length of a consultation, and salary provided by advanced practice nurses in primary care in the United States [27]. In another study, the fee for general medical procedures provided by advanced practice registered nurses in Queensland, Australia was $41 per procedure [28]. In the current study, the cost per chronic disease management service for primary care nurses was estimated at $11.90, which is lower than in the United States and Australia studies. This discrepancy may be due to the different roles, scope, and payment mechanisms of nurses in different countries [27]. In Korea, the annual fee for the services provided by the primary care pilot program for chronic disease management ranges from 352,700 KRW ($272.63) to 389,150 KRW ($300.81) [4]. A local clinic participating in the primary care pilot program for chronic disease management reported that their annual service fee was 200,000 KRW ($154.60) [9]. The average annual number of services provided was 15. The price of one chronic disease management service was 23,513 KRW ($18.18) to 25,943 KRW ($20.05) in the primary care pilot program for chronic disease management, 13,333 KRW ($10.31) in local clinic. Therefore, the current fee for chronic disease management services is converging between approximately 13,333 KRW ($10.31) and 25,943 KRW ($20.05), and the estimated WTP in the current study was within this range. This finding indicates that the estimated WTP in this study reflects the current medical payment systems in Korea. Therefore, the estimated WTP in this study can be used as a basis for determining the prices for chronic disease management services provided by primary care nurses. However, because the current fee for the primary care pilot program for chronic disease management is not the fee separately presented by doctors and nurses [4], the current fee for actual nurse services may be lower than the price suggested by our findings. The estimated WTP may be higher than the actual copay, which may indicate that respondents are willing to pay an additional amount for chronic management services provided by primary care nurses. However, caution is needed in interpreting the results of this study, particularly when the actual fee for chronic management services provided by primary care nurses has not been set.

WTP is the price that consumers intend to pay for the target service. However, the service fee set when the policy is implemented may not match consumers’ WTP because the actual fee for the target service is set to the extent to which the cost of the target service can be calculated and the cost can be guaranteed [29]. Therefore, the actual fee for chronic disease management services provided by primary care nurses will be set to the extent that the cost of chronic disease management services provided by primary care nurses can be calculated and the calculated cost can be guaranteed. In this study, the price that the participants were willing to pay for one chronic disease management service provided by primary care nurses was 15,390.71 KRW ($11.90). If the cost of one chronic disease management service provided by primary care nurses estimated in future studies is more than the 15,390.71 KRW ($11.90 USD) estimated in this study, the government may consider the difference in the amount as a medical care cost (i.e., not charged directly to the recipient).

Participants tended to be unwilling to pay for the services when the bid price was high and when their incomes were low. These results are consistent with those of a study by Lee (2003) who surveyed Seoul citizens aged ≥ 20 years on their WTP for long-term care insurance for the elderly [13]. This finding may indicate that WTP is related to participants’ economic ability to pay. A long-standing limitation of CVM is that WTP is limited by respondents’ ability to pay. In particular, because the hypothetical scenario of this study indicated that primary care nurse services target individuals with chronic diseases, it is possible that participants’ responses reflected their economic ability to pay for services. Therefore, future studies should calculate a more sophisticated and precise value by surveying various individuals’ WTP including those with actual chronic diseases [30].

Several factor affected participants’ WTP. In particular, respondents who were aware of primary care nurses were more willing to pay. Individuals who reported more public awareness and understanding of the role of advanced practice nurses were more aware of the services and medical policies, and showed greater support for the healthcare system [5]. This finding indicates that to set fees for services delivered by primary care nurses, it is necessary to increase public awareness and understanding of primary care nurses. However, only 25.0% of the respondents in this study had heard of primary care nurses indicating a serious lack of awareness of primary care nurses. Thus, efforts are needed at the individual, organization, and government levels to increase public awareness and understanding of primary care nurses and the quality services they provide.

Another interesting finding is that respondents who were presented with high first-bid prices were more likely to be willing to pay for the services. Kim (2009) contended that WTP affects the efficiency of WTP, but does not have a significant effect on estimating WTP [31]. However, a common methodological bias in CVM is that the bid price affects respondents’ WTP [16]. In this study, it was difficult for respondents to estimate the monetary value of the service because the market price of the chronic disease management services provided by primary care nurses has not been set. Therefore, WTP could only be determined based on the suggested bid price presented in the survey. To reduce this bias, the bid prices in this study were selected based on the results of the pilot test. In the pilot study, the distribution of the bid prices was identified first, and then, four bid prices were set in the 15% and 85% range [17]. Nevertheless, because the bid price affected WTP, researchers should pay more attention to the design of a pilot study to identify the bid prices.

The main reason for participants’ unwillingness to pay for chronic disease management services provided by primary care nurses was “The country should provide this service for free.” This response represents people’s expectations that the government should pay for primary care services, and a copay should be set so that all citizens, including low-income individuals, can receive primary care nurse services when needed. However, the government should also try to prevent moral hazard that may occur due to low or no economic burden. Establishing an appropriate copay system prevents unnecessary abuse of medical services, thereby preventing moral hazards due to health insurance. Therefore, it is necessary to carefully set an appropriate copay so the burden of medical expenses is not too small depending on the income level [32].

The second reason for the unwillingness to pay for chronic disease management services provided by primary care nurses was “I am not interested in the chronic disease management services provided by nurses.” This response may be because of a lack of awareness of primary care nurses [33]. Thus, to successfully set the price for primary care nurses in local clinics, the government and organizations must promote the important role of primary care nurses and the services. Given the variability in primary care contexts and perceptions of primary care nurses across countries, it is important to interpret the study results cautiously within the context of each country. However, in countries where the roles of primary care nurses are not yet established, the study results can help policymakers and stakeholders better understand why citizens are either willing or not willing to pay for the services provided by primary care nurses. Nursing services are considered “invisible economics” because they are usually included in the cost of hospitalization rather than billed separately, making them difficult to measure in practice. Thus, few studies have measured the cost of nursing services [34]. The significance of this study lies in our estimation of WTP for nursing services using CVM, which adds methodological evidence for estimating the price for nursing services.

This study has several limitations. First, WTP may have been under- or overestimated. Because WTP was estimated based on a hypothetical scenario, there could be differences between real benefits and what individuals imagine in a hypothetical situation. To reduce this bias, this study complied with the National Oceanic and Atmospheric Administration panel’s recommendations and guidelines for the CVM [16]. The hypothetical scenario was analyzed and modified based on a pilot study and consultations with nurse care coordinators who are currently working at local clinics. However, the estimated WTP should be interpreted with caution. Second, the study participants may not have revealed their exact WTP because they may not have had experience suggesting a preferred price for services. To compensate for these limitations, this study asked two types of questions—DBDC questions first and then open-ended questions—so the participants could estimate their WTP more accurately. Third, this study conducted an online survey, which excluded individuals without Internet access, particularly elderly individuals. To compensate for this limitation, the study sample was constructed considering gender and age, and 19% of the respondents were aged ≥ 60 years in this study. Fourth, the CVM research method relies on participants understanding the hypothetical scenario. However, since this study utilized an online survey, respondents may have had difficulty understanding the scenario.

## Conclusions

This is the first study to estimate the value of chronic disease management services provided by primary care nurses in Korea. With the rapidly aging population in Korea, the disease paradigm is changing from acute to chronic, highlighting the critical need for primary care nurses. Thus, it is timely to conduct a study to determine the economic valuation of primary care nurse services. Additionally, the study participants were recruited nationwide considering gender and age distribution, which increased the generalizability of the study results. In this study, the estimated mean WTP was 15,390.71 KRW ($11.90), and the factors affecting WTP included chronic disease, recognition of primary care nurses, and the first bid price.

This study is meaningful in that it reflects the preferences and values of individuals rather than focusing on service providers that have traditionally been surveyed to estimate fees for medical services. The results of this study are also a good starting point for understanding how individuals perceive and value the benefits of primary care nurse services. In terms of policy, the findings on individuals’ WTP can be used as a basic resource for setting fees for services provided by primary care nurses. It is hoped that the fee for nurse services reflecting the public’s preferences will be set soon to promote the employment of nurses in local clinics and further improve the working environment of primary care nurses.

### Supplementary Information

Additional file 1. Hypothetical scenario

Additional file 2. Estimated process of the willingness to pay for chronic disease management services provided by primary care nurses.

Additional file 3. Econometric estimation using a tobit model.

## Data Availability

Access to the data is restricted because of privacy restrictions. However, the data are available from the corresponding author upon reasonable request.

## Abbreviations

CVM: Contingent valuation method
DBDC: Double-bounded dichotomous choice
KRW: Korean won
MOHW: Ministry of Health and Welfare
OECD: Organization for Economic Co-operation and Development
WTP: Willingness to pay
