# Supplementary material for: Willingness to pay for chronic disease management services provided by primary care nurses
Source: Hum Resour Health. 2024 Jul 8;22:49. doi: 10.1186/s12960-024-00935-8 (PMC11229183; doi:10.1186/s12960-024-00935-8)
Supplement: Supplementary file 1 — Additional file 1. Hypothetical scenario [file 12960_2024_935_MOESM1_ESM.docx]

**Additional file 1. Hypothetical scenario**

Imagine that you are a 68-year-old woman whose children are married and that only you and your husband live in your own homes. You are diagnosed with hypertension 5 years ago and diabetes mellitus 15 years ago. You are taking medications for both hypertension and diabetes mellitus; however, insulin is not needed. You do not measure blood pressure and blood sugar by yourself, and both levels are not controlled. You recently visited the hospital with numbness in your hands and feet and are currently suspected of diabetic peripheral neuropathy. You have no problems regarding your activities of daily living, such as walking, using the bathroom, and preparing meals. You do not perform any exercises, except for walking once or twice a week, due to knee pain. You have an irregular eating habit and frequently experience indigestion. You do not smoke or drink.

In a local clinic, a primary care nurse provides chronic disease management services. If you apply for the chronic disease management services provided by the primary care nurse, you can receive the following services:

- Basic health assessment and evaluation (e.g., height, weight, blood test, urine test, electrocardiogram, and complication test).

- Personalized care plan established with the doctor, primary care nurse, and yourself based on the health assessment.

- Education and counseling about lifestyle, drinking, smoking, exercise, diet, obesity, and chronic disease management, such as self-check of blood pressure and blood test, taking medications, complications, and emergent symptoms.

- Monitoring of your health status and lifestyle via text messages or phone calls.

- Real-time communication via text and call when needed.

- Guidance and link to available community resources.
