# Supplementary figures and images for: Willingness to pay for chronic disease management services provided by primary care nurses
Source: Hum Resour Health. 2024 Jul 8;22:49. doi: 10.1186/s12960-024-00935-8 (PMC11229183; doi:10.1186/s12960-024-00935-8)

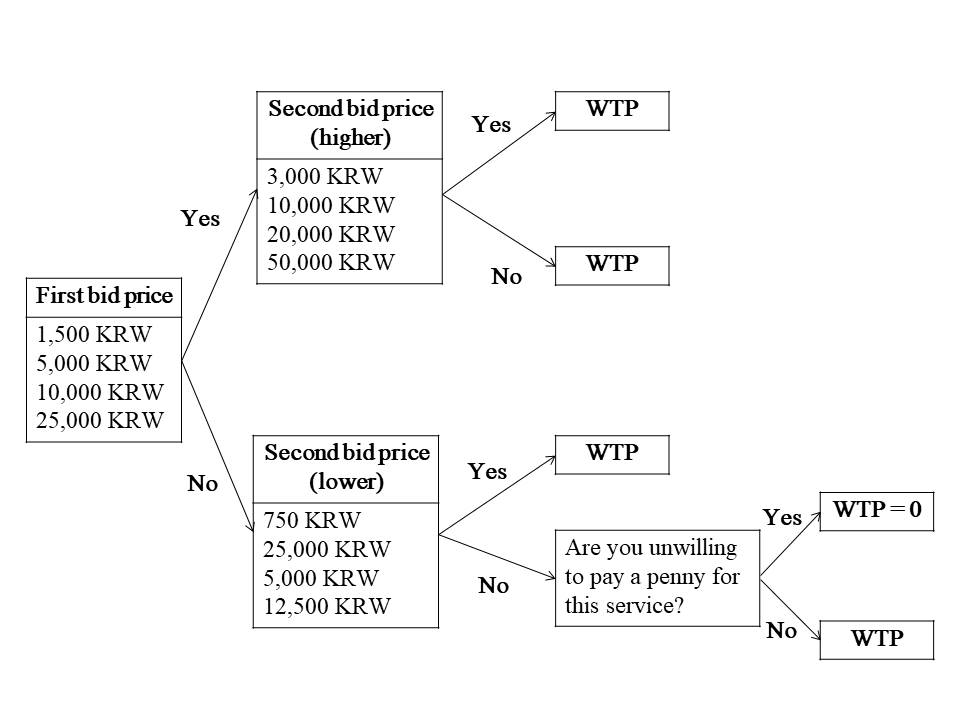

Supplement: Supplementary file 2 — Additional file 2. Estimated process of the willingness to pay for chronic disease management services provided by primary care nurses. [file 12960_2024_935_MOESM2_ESM.jpg]
