# Supplementary material for: Willingness to pay for chronic disease management services provided by primary care nurses
Source: Hum Resour Health. 2024 Jul 8;22:49. doi: 10.1186/s12960-024-00935-8 (PMC11229183; doi:10.1186/s12960-024-00935-8)
Supplement: Supplementary file 3 — Additional file 3. Econometric estimation using a tobit model. [file 12960_2024_935_MOESM3_ESM.docx]

**Additional file 3. Econometric estimation using a tobit model**

The tobit model was expressed by the following relationship:

$y_{i}= x_{i}\beta+ \epsilon_{i}, \epsilon_{i}, \sim N\left( 0, \sigma^{2} \right)$ (1)

$$y_{i}= {y_{i}}^{*} if {y_{i}}^{*}>0$$

$$y_{i}= 0 if {y_{i}}^{*}\leq0$$

In Equation 1, *N* is the number of observations, $y_{i}$ is the observed WTP, ${y_{i}}^{*}$ is the latent variable, $x_{i}$ is the vector of independent variables, $\beta$ is the vector of unknown coefficients, $\sigma$ is a scale parameter, and $\epsilon_{i}$ is an independently distributed error term. The WTP was estimated using maximum likelihood estimation because these data are censored at zero. If the likelihood function of observation is $y_{i}$ = 0, it appears in the form of a distribution function, and if $y_{i}$>0, it appears in the form of a density function.

$\ln L= \sum ln(1-\emptyset(\frac{x_{i}\beta}{\sigma_{u}}))+ \sum\left[ ln\sigma_{u}+ln\emptyset\left( \frac{y_{i}-x_{i}\beta}{\sigma_{u}} \right) \right]$ (2)

Equation 2 is a likelihood function for the entire sample. The coefficient estimate $\beta$ is obtained by maximizing the $\ln L$ obtained by taking the log value of the likelihood function.

Based on Equation 2, Equation 3 of the overall model of the WTP estimation tobit has been applied in this study.

$$y= \alpha+ \beta_{1}Gender+ \beta_{2}Age + \beta_{3}Residences + \beta_{4}Education level+ \beta_{5}Monthly household income + \beta_{6}Marital status + \beta_{7}Currently working + \beta_{8}Subjective health status + \beta_{9}Chronic disease + \beta_{10}Social activity + \beta_{11}Recognition of primary care nurse + \beta_{12}Recognition of primary care pilot program for chronic diseases management + \beta_{13}First bid (3)$$
